# Analysis of the dietary factors associated with suspected pediatric nonalcoholic fatty liver disease and potential liver fibrosis: Korean National Health and Nutrition Examination Survey 2014-2017

**DOI:** 10.1186/s12887-020-02022-y

**Published:** 2020-03-14

**Authors:** Mi Jin Kim, Kyung Jae Lee

**Affiliations:** Department of Pediatrics, Hallym University Sacred Heart Hospital, Anyang, Republic of Korea

**Keywords:** Non-alcoholic fatty liver disease, Children, Diet

## Abstract

**Background:**

The prevalence of nonalcoholic fatty liver disease (NAFLD) has increased as the obese pediatric population has increased. NAFLD causes progressive liver injury and the only effective treatment is lifestyle modifications. However, few studies have examined the dietary risk factors for pediatric NAFLD or liver fibrosis. Here, we evaluated the dietary factors associated with suspected NAFLD and potential liver fibrosis in Korean children.

**Methods:**

Data collected from 1674 children and adolescents aged 10–18 years during the 2014–2017 Korean National Health and Nutrition Examination Surveys analyzed. The 24-h recall method measured the food consumed 1 day before the survey. The “suspected NAFLD” group included excessive body mass index (BMI) subjects ≥ 85th percentile) with alanine aminotransferase (ALT) levels exceeding the upper normal limit (24.1 U/L for boys and 17.7 U/L for girls); the “healthy control” group included subjects with a BMI and ALT level below these thresholds. Sodium intake was assessed by the urinary sodium-to-urinary specific gravity unit ratio (U-Na-to-SGU ratio). A pediatric NAFLD index (PNFI) higher than 3 indicated potential liver fibrosis.

**Results:**

The overall prevalence of suspected NAFLD and potential liver fibrosis was 8.2 and 4.5%, respectively. The suspected NAFLD group had a larger proportion of males and subject with a greater height, BMI standard deviation score (BMI-SDS), systolic and diastolic blood pressure SDS, waist circumference, hemoglobin A1c, and levels of total cholesterol, triglycerides, aspartate aminotransferase (AST) and ALT than the control group. The suspected NAFLD group presented significantly higher U-Na-to-SGU ratios and cholesterol intake. The PNFI > 3 subgroup included a significantly larger proportion of males and subjects with higher BMI-SDS, AST and ALT values, and intake of water, carbohydrate, protein, calcium, phosphorus, iron and vitamin B2. After adjusting for confounders, male, BMI-SDS, AST, and protein and carbohydrate intake were independent risk factors for potential liver fibrosis. Niacin intake was an independent protective factor for potential liver fibrosis.

**Conclusions:**

Children with suspected NAFLD had higher urinary sodium level and cholesterol intake than healthy controls. Protein and carbohydrate intake were independent risk factors for potential liver fibrosis; niacin was an independent protective factor.

## Background

Pediatric nonalcoholic fatty liver disease (NAFLD) is defined as chronic hepatic steatosis in children (18 years or younger) that is not secondary to a genetic/metabolic disease, infection, use of steatogenic medications, ethanol consumption, or malnutrition [1]. NAFLD is an inclusive term referring to the full spectrum of diseases from fatty infiltration of the liver, typically more than 5% of the liver analyzed using imaging, direct quantification, or histological estimation [1]. Because obesity is strongly correlated with NAFLD, and the obese pediatric population has increased, the prevalence of NAFLD has increased and has become the most common cause of chronic pediatric liver disease in developed countries [2]. In Korea, the estimated prevalence of adolescents with NAFLD also increased from 4.7% in 2010 to 5.9% in 2015, and an increased ALT level was associated with the male sex, obesity and truncal obesity [3].

NAFLD tends to progress and could transit into the adult period; therefore, an early diagnosis and treatments are important [4]. Although the data for the natural history of pediatric NAFLD are limited, pediatric NAFLD appears to be a more severe phenotype than the disease in adults, and 15% of the children with NALFD have stage 3 fibrosis or higher at diagnosis [5, 6].

The gold standard of treatment for NAFLD is a nonpharmacological intervention, such as weight reduction and physical exercise [7–9]. Therefore, nutrition is a key factor that affects the progression and development of NAFLD. Some efforts have attempted to reveal the relationship between dietary factors and fatty liver or liver fibrosis in adult populations [10–19]. Although overnutrition is the main cause of NAFLD, each nutrient may function as a causative or protective factor. Fat- and carbohydrate-rich diets contribute to the pathogenesis of NAFLD [10, 14, 16, 20]. In contrast, fiber and low-glycemic-index diets, as well as monounsaturated fatty acids (MUFAs) and omega-3 fatty acids, exert protective effects on NAFLD [11, 14, 16].

Sodium intake positively correlates with metabolic syndrome and hypertension in children, adolescents and adults [21–23]. One study revealed an independent association between high sodium intake and an increased risk of NAFLD and advanced liver fibrosis in healthy Korean adults [19]. However, to date, only a few studies have examined the dietary risk factors for NAFLD or liver fibrosis in children and adolescents [24–26].

In this study, we attempted to identify the dietary factors that affect suspected NAFLD in Korean children, and we further evaluated the factors associated with potential liver fibrosis in children with suspected NAFLD.

## Methods

### Study participants

**Fig. 1:**
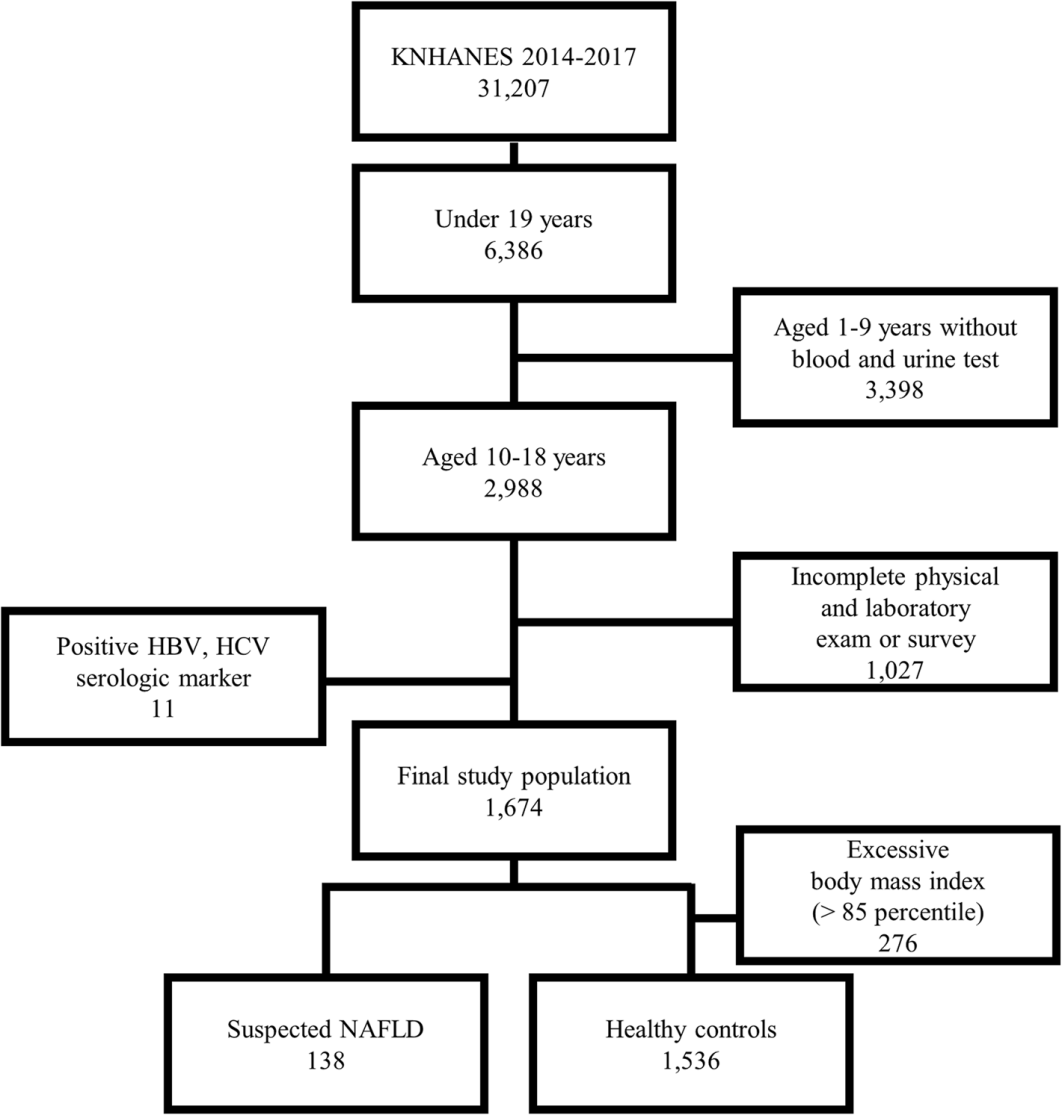
Flow chart showing the selection of the sample analyzed in this study. In total, 1674 Korean children were included in the present study

The data used in this study were obtained from the 2014–2017 Korean National Health and Nutrition Examination Surveys (KNHANES). The KNHANES is a cross-sectional, nationwide, representative survey composed of a health interview survey, a nutrition survey, and a health examination survey that is performed periodically by the Division of Chronic Disease Surveillance, Korean Centers for Disease Control and Prevention [27]. Data were collected through household interviews and direct, standardized physical examinations conducted in mobile examination centers [19]. Out of 31,207 participants, 6386 were under the age of 19. Only 2988 children and adolescents aged 10–18 years were included in this study as blood tests and urine tests were conducted only on children over the age of 10. We excluded 11 subjects with positive serological markers for hepatitis B or hepatitis C viruses. and 1027 subjects who did not complete the physical and laboratory examinations or nutrition survey. We divided our study participants into two groups: “suspected NAFLD” and “healthy control” groups. We defined the “suspected NAFLD” group as participants who had an excessive body mass index (BMI) above the 85th percentile and had alanine aminotransferase (ALT) levels higher than the upper normal limit (24.1 U/L for boys and 17.7 U/L for girls) [28]. We also defined the “healthy control” group as participants who had a BMI below the 85th percentile and an ALT level below the normal limit. Additionally, we excluded 276 participants with an excessive BMI above the 85th percentile from the healthy control group because they had a potential risk of NAFLD, although their ALT level were normal. One thousand six hundred seventy-four participants were included in the suspected NAFLD (*n* = 138) and healthy control groups (*n* = 1536) in the present study. Figure 1 shows the process used to select participants for this study.

The ALT cutoff value for NAFLD varies according to the age, sex and population. In the North American Society of Pediatric Gastroenterology, Hepatology and Nutrition clinical guideline, 2 times the sex-specific ALT level (ALT ≥ 50 for boys and ≥ 44 for girls) observed in overweight and obese children age 10 years or older is used to diagnose NAFLD [1]. However, the Screening ALT Elevation in Today’s Youth (SAFETY) study suggested that the current upper normal limit of the ALT level is too high to detect chronic liver diseases, including NAFLD; therefore, they suggested normal ALT levels of 25.8 U/L for boys and 22.1 U/L for girls among the 95th percentile for healthy weight, metabolically normal, liver disease-free pediatric participants [29]. Welsh JA et al. defined suspected NAFLD as overweight (BMI > 85th percentile) plus elevated ALT levels (boys > 25.8 U/L and girls > 22.1 U/L) in the National Health and Examination Survey conducted in the US [30]. The purpose of this study was to screen children with suspected NAFLD and identify related dietary factors. Thus, we used sex-specific cutoff values for ALT levels that were recently proposed for the Korean pediatric population [28].

The study was approved by the Ethics Committees of Hallym University Sacred Heart Hospital (IRB no. 2019–11-031) and KNHANES surveys are approved by the Korean Center for Disease Control and KNHANES participants and legal representatives of children under 14 years provided written informed consent. The data from the KNHANES surveys are available at http://knhanes.cdc.go.kr/.

### Anthropometric measurements

Trained examiners performed the anthropometric and biochemical measurements. Height (cm) and weight (kg) were measured to the nearest 0.1 cm using a Seca 225 instrument (Seca, Hamburg, Germany) and 0.1 kg using a GL-6000-20 scale (G-tech, Seoul, Korea), respectively, when the subjects were wearing the examination gown without personal belongings. The BMI was calculated as the weight (kg)/square of height (m^2^), and the standard deviation score (SDS) for BMI was used to correct the uneven distributions between different age groups. We used the 2017 Korean national growth chart for children and adolescents developed from the growth reference data [31]. WC was measured at the midline between the lower rib margin and the iliac crest to the nearest 0.1 cm. Systolic blood pressure (SBP) and diastolic blood pressure (DBP) were measured three times on the right upper arm at 2-min intervals with a mercury sphygmomanometer (Baumanometer; W.A. Baum Co., Copiague, NY), and the mean values of the second and third blood pressure measurements were used in this study. Blood pressure SDS, which measures the relative blood pressure adjusted for the child’s age, sex and height SDS, was analyzed in the current study. Hypertension was defined as SBP or DB*P* values that were greater than or equal to the 95th percentile of blood pressure adjusted for age, sex and height [32].

### Laboratory tests

Blood and urine samples were randomly collected after an 8-h fasting. Then, these samples were immediately processed, refrigerated, and transported to the central laboratory (Neodin Medical Institute, Seoul, Korea). Routine biochemistry tests, including blood glucose, total cholesterol, triglycerides (TGs), aspartate aminotransferase (AST), ALT, urinary sodium (U-Na) and urinary specific gravity (U-SG) levels, were measured using a Hitachi 7600 automatic analyzer (Hitachi, Tokyo, Japan). We considered subjects to have hypercholesterolemia if their cholesterol level was greater than or equal to 200 mg/dL and hypertriglyceridemia if their triglyceride level was greater than or equal to 130 mg/dL [33].

### Nutritional intake assessment

The type and quantity of food consumed 1 day before the survey was measured by 24-h recall method with dietary questionnaires. We used the reference daily intake for Koreans 2015 from the Ministry of Health and Welfare, the Korean Nutrition Society to compare the dietary intake values [34].

Because the answers recorded on dietary recall questionnaires might be incorrect, the 24-h urinary sodium level is usually measured to estimate the exact intake of sodium [35]. However, a 24-h urine sample is difficult to collect, and the cost is relatively expensive. One method for measuring sodium intake is a spot urine sodium level calculated from the urinary sodium (U-Na) levels corrected by specific gravity (SG) or the urinary creatinine level [21, 36, 37]. In the present study, we used the calculated parameter (SG − 1) × 100 as an SG unit (SGU) [21, 36, 37].

$$ \frac{\mathrm{Urinary}\ \mathrm{sodium}\ \left(\mathrm{U}-\mathrm{Na}\right)}{\mathrm{Specific}\ \mathrm{gravity}\ \mathrm{unit}\ \left(\mathrm{SGU}\right)} $$

$$ \mathrm{SGU}=\left(\mathrm{Urinary}\ \mathrm{specific}\ \mathrm{gravity}-1\right)\times 100 $$

### Fibrosis index

In the present study, the pediatric NAFLD fibrosis index (PNFI) was used to predict potential liver fibrosis in children and adolescents [38]. Based on the literature, the suspected NAFLD group was divided into two subgroups based on the PNFI. Patients with a PNFI greater than 3 (PNFI > 3) were considered to have potential liver fibrosis, and patients with a PNFI of 3 or less (PNFI ≤ 3) were not considered to have fibrosis [38–41].

$$ \mathrm{PNFI}=\frac{1}{1+{\mathrm{e}}^{- lp}}\times 10 $$

$$ lp=-6.539\times {\log}_{\mathrm{e}}\left[\mathrm{age}\ \left(\mathrm{years}\right)\right]+0.207\times \mathrm{waist}\ \left(\mathrm{cm}\right)+1.957\times {\log}_{\mathrm{e}}\left[\mathrm{triglycerides}\ \left(\frac{\mathrm{mg}}{\mathrm{dl}}\right)\right]-10.074 $$

### Statistical analysis

Statistical analyses were performed using PASW statistics software (SPSS version 23.0, IBM SPSS Inc., Chicago, IL, USA). The characteristics of participants according to the presence of suspected NAFLD or potential liver fibrosis were compared using independent-sample Student’s t-tests and chi-square tests for categorical measures. We also analyzed the characteristics of the participants after stratification by age and gender using independent-sample Student’s t-tests and the Mann-Whitney U test if the data did not display a normal distribution. We performed a multivariate logistic regression analysis and estimated and the odds ratio (OR) and 95% confidence intervals (CI) by adjusting for generally known confounding factors and statistically significant factors in this study to investigate the associations between dietary factors and potential liver fibrosis. All data are presented as means ± standard errors (SEs) or percentages (%) for categorical variables. *P* values < 0.05 were considered statistically significant.

## Results

The overall prevalence of suspected NAFLD was 8.2% in the current study. The characteristics of the study participants according to the presence of suspected NAFLD are presented in Table 1. The suspected NAFLD group exhibited a significantly higher height (*P* < 0.001), BMI-SDS (*P* < 0.001), SBP-SDS (*P* < 0.001), DBP-SDS (*P* < 0.001), WC (*P* < 0.001), total cholesterol level (*P* < 0.001), TG level (*P* < 0.001), AST level (*P* < 0.001), ALT level (*P* < 0.001) and fasting glucose level (*P* = 0.039) than the healthy control group.

**Table 1:** Characteristics of study participants according to the presence of suspected NAFLD in Korean children and adolescents aged 10–18 years

		Suspected NAFLD	Healthy controls	*P* value
		(*n* = 138, 8.2%)	(*n* = 1536, 91.8%)	
Age		14.34 ± 0.21	13.77 ± 0.06	0.011
Sex	Male (*n* = 932)	92 (66.7%)	840 (54.7%)	0.007
	Female (*n* = 742)	46 (33.3%)	696 (45.3%)	
Ht (cm)		163.96 ± 0.87	160.01 ± 0.29	< 0.001
BMI (kg/m^2^)		28.12 ± 0.31	19.29 ± 0.06	< 0.001
BMI-SDS		2.35 ± 0.08	−0.49 ± 0.02	<0.001
Hypertension (n, %)		47 (34.1%)	221 (14.4%)	<0.001
SBP (mmHg)		115.70 ± 0.91	107.16 ± 0.24	<0.001
SBP-SDS		0.33 ± 0.08	−0.27 ± 0.02	<0.001
DBP (mmHg)		69.99 ± 0.77	65.28 ± 0.22	<0.001
DBP-SDS		1.08 ± 0.09	0.52 ± 0.03	<0.001
WC (cm)		89.35 ± 0.85	66.60 ± 0.18	<0.001
Hemoglobin A1c (%)		5.51 ± 0.06	5.37 ± 0.01	0.023
Hypercholesterolemia (n, %)		26 (18.8%)	135 (8.8%)	<0.001
Total cholesterol (mg/dL)		174.22 ± 2.38	161.39 ± 0.68	<0.001
Hypertriglyceridemia (n, %)		38 (27.5%)	178 (11.6%)	<0.001
TGs (mg/dL)		113.68 ± 5.17	81.42 ± 1.28	<0.001
AST (IU/L)		32.41 ± 2.50	19.32 ± 0.17	<0.001
ALT (IU/L)		49.64 ± 4.52	13.37 ± 0.32	<0.001
Fasting glucose (mg/dL)		94.56 ± 1.44	91.53 ± 0.18	0.039
U-Na-to-SGU ratio		55.47 ± 1.96	51.21 ± 0.56	0.029

*Ht* Height, *BMI* Body mass index, *SDS* Standard deviation score, *SBP* Systolic blood pressure, *DBP* Diastolic blood pressure, *WC* Waist circumference, *TGs* Triglycerides, *AST* Aspartate aminotransferase, *ALT* Alanine aminotransferase, *U-Na* Urinary sodium, *SGU* Specific gravity unit

The U-Na-to-SGU ratio was significantly higher in the suspected NAFLD group than in the healthy control group (55.47 ± 1.96 vs. 51.21 ± 0.56, *P* = 0.029). Higher dietary sodium intake was also recorded in the suspected NAFLD group, but the difference was not significant (3692.58 ± 195.47 vs. 3462.63 ± 51.32, *P* = 0.204, Table 2**).** In this population, the sodium intake of both groups was much higher than the recommended reference daily intake. Regarding the other dietary factors, only cholesterol intake was noticeably different between the suspected NAFLD group and the control group (366.60 ± 27.13 vs. 302.11 ± 6.10, *P* = 0.022, Table 2). We also compared dietary intake to the recommended reference daily intake between the suspected NAFLD group and control group after stratification by gender and age. For boys, overall dietary intake was higher in the potential NAFLD group. The intake of cholesterol (412.19 ± 36.78 vs. 334.32 ± 9.04, *P* = 0.042) and polyunsaturated fatty acids (PUFAs; 17.51 ± 1.48 vs. 14.10 ± 0.33, *P* = 0.027) was significantly higher in boys with suspected NAFLD. Total water intake was significantly higher in healthy controls than participants with suspected NAFLD among girls aged 12–18 years (1723.86 ± 81.81 vs. 1909.79 ± 28.16, *P* = 0.003).

**Table 2:** Dietary factors of study participants according to the presence of suspected NAFLD in Korean children and adolescents aged 10–18 years and reference daily intake

	Suspected NAFLD	Healthy controls	*P* value	Reference daily intake
Total energy intake (kcal)	2214.74 ± 90.84	2172.17 ± 23.01	0.601	1900 ~ 2700
Total water intake (g)	953.76 ± 48.12	960.57 ± 14.02	0.891	900 ~ 2600
Carbohydrate intake (g)	311.65 ± 12.79	323.14 ± 3.26	0.319	247.5 ~ 438.75
Protein intake (g)	87.99 ± 4.81	79.16 ± 1.15	0.076	40 ~ 65
Fat intake (g)	64.90 ± 3.70	61.40 ± 1.00	0.320	15 ~ 30
Cholesterol intake (mg)	366.60 ± 27.13	302.11 ± 6.10	0.022	< 300
SFA intake (g)	20.18 ± 1.19	20.48 ± 0.36	0.807	16 ~ 24
MUFA intake (g)	20.96 ± 1.33	20.36 ± 0.37	0.647	
PUFA intake (g)	14.92 ± 1.07	12.95 ± 0.24	0.074	
Fiber intake (g)	18.77 ± 0.89	19.05 ± 0.29	0.785	20 ~ 25
Calcium intake (mg)	514.38 ± 34.39	513.40 ± 8.19	0.973	650 ~ 1000
Phosphorus intake (mg)	1155.95 ± 48.45	1119.61 ± 13.34	0.438	1000 ~ 1200
Iron intake (mg)	15.61 ± 0.94	15.65 ± 0.53	0.981	7 ~ 16
Sodium intake (mg)	3692.58 ± 195.47	3462.63 ± 51.32	0.204	1400 ~ 1500
Potassium intake (mg)	2686.12 ± 116.95	2628.61 ± 33.49	0.623	3000 ~ 3500
Vitamin A intake (ngE)	611.75 ± 53.50	627.55 ± 36.37	0.897	550 ~ 850
Vitamin B1 intake (mg)	1.94 ± 0.10	1.90 ± 0.03	0.753	0.9 ~ 1.3
Vitamin B2 intake (mg)	1.66 ± 0.09	1.55 ± 0.02	0.214	1.0 ~ 1.7
Vitamin C intake (mg)	65.69 ± 6.46	72.01 ± 2.19	0.403	70 ~ 105
Niacin intake (mg)	16.21 ± 0.85	15.39 ± 0.23	0.310	12 ~ 16

*SFAs* Saturated fatty acids, *MUFAs* Monounsaturated fatty acids, *PUFAs* Poly-unsaturated fatty acids

The characteristics of the participants in the suspected NAFLD subgroup according to PNFI are shown in Table 3. In addition to age, WC, and TG levels, which are the variables necessary for measuring PNFI, a significantly larger proportion of subjects in the PNFI > 3 group were male and significantly larger proportions exhibited a higher BMI, BMI-SDS, AST and ALT levels than participants in the NAFLD without liver fibrosis group (*P* < 0.001). Regarding nutritional factors, total water intake (*P* = 0.004), carbohydrate intake (*P* = 0.034), protein intake (*P* = 0.021), calcium intake (*P* = 0.015), phosphorus intake (*P* = 0.048), iron intake (*P* = 0.006) and vitamin B2 intake (*P* = 0.015) were higher in the PNFI > 3 group (Table 4). We also compared dietary intake to the recommended reference dietary intake between the PNFI≤3 group and PNFI>3 group after stratification by gender and age. Higher niacin intake was recorded in all age subgroups of the PNFI≤3 group among girls, but the difference was not statistically significant (14.29 ± 1.21 vs. 13.13 ± 1.57, *P* = 0.554).

**Table 3:** Characteristics of the participants in the suspected nonalcoholic fatty liver disease group according to the pediatric NAFLD fibrosis index

		PNFI≤3	PNFI> 3	*P* value
		(*n* = 49, 35.5%)	(*n* = 89, 64.5%)	
Age (y)		15.16 ± 0.33	13.89 ± 0.26	0.003
Sex	Male (*n* = 92)	23 (46.9%)	69 (77.5%)	<0.001
	Female (*n* = 46)	26 (53.1%)	20 (22.5%)	
Ht (cm)		163.40 ± 1.29	164.28 ± 1.16	0.634
BMI (kg/m^2^)		26.74 ± 0.37	28.87 ± 0.41	<0.001
BMI-SDS		1.97 ± 0.10	2.57 ± 0.10	<0.001
SBP (mmHg)		114.67 ± 1.63	116.27 ± 1.11	0.403
SBP-SDS		0.26 ± 0.15	0.36 ± 0.08	0.481
DBP (mmHg)		69.18 ± 1.31	70.43 ± 0.97	0.445
DBP-SDS		0.93 ± 0.17	1.17 ± 0.11	0.229
WC (cm)		82.17 ± 0.82	93.30 ± 1.03	<0.001
Platelet count (×10^3^ /μL)		312.41 ± 9.01	320.93 ± 6.34	0.433
Hemoglobin A1c (%)		5.42 ± 0.05	5.56 ± 0.09	0.289
Total cholesterol (mg/dL)		171.73 ± 3.97	175.60 ± 2.98	0.439
TGs (mg/dL)		85.57 ± 5.89	129.16 ± 6.81	<0.001
AST (IU/L)		23.65 ± 0.97	37.24 ± 3.75	<0.001
ALT (IU/L)		32.73 ± 2.07	58.96 ± 6.73	<0.001
Fasting glucose (mg/dL)		92.65 ± 1.65	95.61 ± 2.04	0.329
U-Na-to-SGU ratio		50.95 ± 3.69	57.95 ± 2.22	0.087

*Ht* Height, *BMI* Body mass index, *SDS* Standard deviation score, *SBP* Systolic blood pressure, *DBP* Diastolic blood pressure, *WC* Waist circumference, *TGs* Triglycerides, *AST* Aspartate aminotransferase, *ALT* Alanine aminotransferase, *U-Na* Urinary sodium, *SGU* Specific gravity unit

**Table 4:** Dietary factors of the suspected nonalcoholic fatty liver disease group according to the pediatric NAFLD fibrosis index and reference daily intake

	PNFI≤3	PNFI >3	*P*	Reference daily intake
Total energy intake (kcal)	1986.38 ± 112.07	2340.47 ± 125.06	0.062	1900 ~ 2700
Total water intake (g)	788.37 ± 56.15	1044.98 ± 66.15	0.004	900 ~ 2600
Carbohydrate intake (g)	275.14 ± 15.96	331.75 ± 17.48	0.034	247.5 ~ 438.75
Protein intake (g)	75.23 ± 4.91	95.01 ± 6.87	0.021	40 ~ 65
Fat intake (g)	59.03 ± 4.46	68.13 ± 5.17	0.185	15 ~ 30
Cholesterol intake (mg)	317.86 ± 32.99	393.43 ± 37.78	0.184	< 300
SFA intake (g)	18.90 ± 1.53	20.89 ± 1.64	0.424	16 ~ 24
MUFA intake (g)	19.24 ± 1.70	21.91 ± 1.84	0.340	
PUFA intake (g)	12.98 ± 1.20	15.99 ± 1.51	0.179	
Fiber intake (g)	17.36 ± 1.48	19.55 ± 1.11	0.241	20 ~ 25
Calcium intake (mg)	417.31 ± 37.18	567.82 ± 48.46	0.015	650 ~ 1000
Phosphorus intake (mg)	1039.88 ± 60.58	1219.85 ± 66.59	0.048	1000 ~ 1200
Iron intake (mg)	12.56 ± 1.05	17.29 ± 1.31	0.006	7 ~ 16
Sodium intake (mg)	3321.73 ± 296.53	3896.75 ± 253.96	0.160	1400 ~ 1500
Potassium intake (mg)	2379.48 ± 148.32	2854.94 ± 159.68	0.051	3000 ~ 3500
Vitamin A intake (ngE)	499.98 ± 57.20	673.29 ± 76.20	0.071	4550 ~ 850
Vitamin B1 intake (mg)	1.77 ± 0.14	2.03 ± 0.13	0.194	0.9 ~ 1.3
Vitamin B2 intake (mg)	1.38 ± 0.11	1.82 ± 0.12	0.015	1.0 ~ 1.7
Vitamin C intake (mg)	63.98 ± 10.98	66.63 ± 8.03	0.845	70 ~ 105
Niacin intake (mg)	15.09 ± 2.88	16.82 ± 1.22	0.251	12 ~ 16

*PNFI* Pediatric NAFLD fibrosis index, *SFAs* Saturated fatty acids, *MUFAs* Monounsaturated fatty acids, *PUFAs* Poly-unsaturated fatty acids

The adjusted ORs of risk factors for potential liver fibrosis are presented in Table 5.After adjusting for confounding factors, males had an 8.036-fold higher risk of potential liver fibrosis (*P* < 0.001) than females in the NAFLD group. As BMI-SDS and AST levels were increased, the risk of potential liver fibrosis also increased independently (BMI-SDS: OR 3.321, *P* < 0.001, AST: OR 1.059, *P* = 0.022). Regarding dietary factors, protein and carbohydrate intake increased the risk of potential liver fibrosis (protein intake: OR 1.053. *P* = 0.007, carbohydrate intake: OR 1.028, *P* = 0.049). Niacin intake exerted a protective effect on potential liver fibrosis (OR 0.862, *P* = 0.019).

**Table 5:** Adjusted ORs (95% CI) of risk factors for potential liver fibrosis in Korean children and adolescents aged 10–18 years with suspected nonalcoholic fatty liver disease

	Adjusted OR (95% CI)	*P* value
Sex (1: male, 2: female)	8.036 (2.62–24.61)	<0.001
BMI-SDS	3.321 (1.75–6.31)	<0.001
AST	1.059 (1.01–1.11)	0.022
Protein intake	1.053 (1.01–1.09)	0.007
Carbohydrate intake	1.028 (1.00–1.06)	0.049
Niacin intake	0.862 (0.76–0.98)	0.019

*OR* Odds ratio, *BMI-SDS* Standard deviation score for the body mass index, *AST* Aspartate aminotransferase

## Discussion

To our knowledge, few published studies have examined dietary factors associated with NAFLD and fibrosis in a pediatric population, although fibrosis is the most important concern of NAFLD. Therefore, we performed this study to analyze the dietary factors associated with suspected pediatric NAFLD and potential liver fibrosis.

Children with suspected NAFLD had higher U-Na-to-SGU ratios and cholesterol intake than healthy controls. High dietary sodium intake is a well-known risk factor for metabolic syndrome and hypertension [21, 42, 43], and even NAFLD in the adult population [18, 19, 44]. However, data on the association between sodium intake and pediatric NAFLD are lacking. Along with metabolic syndrome, hypertension and diabetes, NAFLD is one of the most important complications of obesity. Our result is clinically meaningful to confirm the harmful effects of a high level of dietary sodium intake in children. The average sodium intake of both the suspected NAFLD and healthy control groups was higher than the recommended reference daily intake, which has been widely reported in studies of adult subjects [45, 46]. The possible underlying cause is the Korean preference for high sodium food such as soup, ramen, pickled food and kimchi [45, 46].

Although the gold standard for the assessment of sodium intake is a 24-h urine collection, a spot urine sample is widely used in large population studies such as KNHANES and in the clinic because of its convenience. Furthermore, an applicable formula for estimating 24-h urinary sodium excretion in children is unavailable. Based on other studies, we used the U-Na-to-SGU ratio as a surrogate marker of sodium intake [21, 36, 37].

In our study, significantly higher cholesterol intake was observed in the suspected NAFLD group than in the healthy controls. Fat intake is a well-known risk factor for hepatic steatosis, but some types of fat may prevent the development of NAFLD. MUFAs and PUFAs, including omega-3 fatty acids, protect against NAFLD by increasing fatty acid oxidation and reducing de novo lipogenesis [13, 47], whereas saturated fatty acids (SFAs) induce hepatocyte injury [20]. Cholesterol intake was significantly higher and PUFA intake was significantly lower in nonobese patients with NAFLD than in obese with NAFLD [44]. Therefore, he consumption of MUFA-rich foods (nuts, olive oil and avocado) and PUFA-rich foods (sea fish and green leafy vegetables) rather than SFA-rich foods (meats and dairy products) might improve NAFLD [16]. However, in the current study, PUFA intake was significantly higher in boys with suspected NAFLD. As the overall dietary intake of boys with suspected NAFLD was higher than healthy boys, we postulated that overnutrition potentially explained the differences in the results.

We also investigated the factors associated with liver fibrosis, which is the most important clinical issue for patients with NAFLD. Although a liver biopsy is the most accurate way to confirm fibrosis, because of its invasiveness, noninvasive markers such as the PNFI, fibrosis-4 index (FIB-4), AST-to-platelet ratio index (APRI) and pediatric NAFLD fibrosis score are usually used [41, 48]. Because the subjects included in the KNHANES data were young adolescents, only a few patients with significant fibrosis were identified in this population based on the FIB-4 and APRI. The PNFI is commonly used in patients with pediatric NAFLD to indirectly confirm the presence of liver fibrosis [38–41]. One study performed in 111 Italian children with NAFLD showed that PNFI value less than 3 points, confidently excluded fibrosis with a high sensitivity (93.4%) [39]. Another pediatric study evaluated the rs641738 polymorphism in the membrane-bound O-acyltransferase domain containing the protein 7 gene, which is associated with an increased risk of NAFLD. The authors also defined a PNFI score between 3 and 8.9 points as a potential marker of early fibrosis [38]. We also used a PNFI cutoff scorer of 3 points to distinguish subjects with potential fibrosis from subjects without fibrosis.

Protein and carbohydrate intake were risk factors for potential liver fibrosis. This finding is supported by previous studies showing that the ingestion of a diet excessively rich in carbohydrates, particularly fructose, causes de novo lipogenesis, which may induce hepatic fat accumulation [10, 49]. The consumption of sugar-sweetened beverages is associated with metabolic syndrome and fatty liver in adult and pediatric patients [50, 51]. In contrast, protein intake exerts a positive effect on NAFLD by reducing fat deposition and plasma cholesterol levels [17, 20]. According to the results of an animal study, a high-protein and low-carbohydrate diet prevents hepatic steatosis by reducing de novo lipogenesis [52]. However, unlike previous studies, protein intake increased the risk of liver fibrosis in our study. Although each nutrient plays its own role in NAFLD, overnutrition is the main problem of patients with NAFLD, which might explain the different findings.

In addition, niacin intake was a protective factor against potential liver fibrosis. Niacin is one of the vitamin B complexes that is present in tuna, mushrooms, peanuts, avocado, and green peas. Although the precise mechanism is not completely known, niacin inhibits lipolysis by acting on the hydroxy-carboxylic acid receptor 2 in peripheral adipose tissue, ultimately reducing the reflux of free fatty acids to the liver [53]. Recent studies reported beneficial effects of a higher level of niacin intake on improving hepatic steatosis in patients with NAFLD [12, 54]. In one randomized controlled trial of 39 patients treated with niacin for 23 weeks, the liver fat content decreased significantly after treatment [55]. Moreover, a mechanistic study comparing human hepatocytes treated with niacin to untreated control hepatocytes found that niacin prevented fat accumulation in hepatocytes by reducing hepatocyte diacylglycerol acyltransferase 2 and NADPH oxidase activity [15]. However, the protective effect of niacin should be interpreted with caution until it has been confirmed in a large-scale study.

The present study has several limitations. First, the causal relationship between dietary factors and NAFLD or liver fibrosis cannot be proven because this study employs a cross-sectional design. Second, as nutritional surveys are based on 24-h dietary recall, differences between actual nutritional intake and the survey responses might exist, depending on the participant’s memory. Third, although we used the U-Na-to-SGU ratio to estimate sodium intake, daily variability may exist. Fourth, the study defined suspected NAFLD and potential liver fibrosis using a noninvasive method without hepatic imaging or biopsies.

To our knowledge, this epidemiological study is the first to evaluate the various dietary factors associated with suspected NAFLD and potential fibrosis in the general pediatric population. Although our results were unable to provide strong evidence because of some limitations of the study, our study produced some meaningful results. Because of a lack of data, very limited dietary guidance is available to provide to patients with NAFLD. We expect that the current study will be a useful reference for further studies on dietary factors and may be helpful for developing dietary guidelines for children with NAFLD in the future.

## Conclusions

In the current study, children with suspected NAFLD had higher urinary sodium and cholesterol intake than healthy controls. Protein and carbohydrate intake were independent risk factors for potential liver fibrosis; niacin intake was an independent protective factor.

## Data Availability

The data from KNHANES surveys are available at http://knhanes.cdc.go.kr/.

## Abbreviations

NAFLD: Nonalcoholic fatty liver disease
KNHANES: Korean National Health and Nutrition Examination Surveys
WC: Waist circumference
BMI: Body mass index
SDS: Standard deviation score
SBP: Systolic blood pressure
DBP: Diastolic blood pressure
TGs: Triglycerides
AST: Aspartate aminotransferase
ALT: Alanine aminotransferase
U-Na: Urinary sodium
SGU: Specific gravity unit
MUFAs: Monounsaturated fatty acids
PUFAs: Polyunsaturated fatty acids
SFAs: Saturated fatty acids
PNFI: Pediatric NAFLD index
FIB-4: Fibrosis-4 index
APRI: AST-to-platelet ratio index
